# Supplementary material for: Changes in sucrose metabolism in maize varieties with different cadmium sensitivities under cadmium stress
Source: PLoS One. 2020 Dec 11;15(12):e0243835. doi: 10.1371/journal.pone.0243835 (PMC7732117; doi:10.1371/journal.pone.0243835)
Supplement: Attachment — Submitted filename: Response to reviewers.docx [file pone.0243835.s002.docx]

Dear Editor

We would like to thank you for your letter and for the reviewers’ comments and constructive suggestions about our manuscript entitled “Changes in the sucrose metabolism of maize varieties with different Cd sensitivities under Cd stress” (ID: PONE-D-20-20550).

We found that your comments were useful and helped us improve our manuscript. We have carefully considered your comments and have revised the manuscript to incorporate your suggestions. The revised parts are marked in red in the paper. We have compiled the main corrections in the paper and the responses to the reviewers’ comments below. We hope that the corrections will meet your approval. Again, we would like to express our appreciation for your comments and we look forward to hearing from you in due course.

Responses to the reviewers’ comments:

Reviewer #1:

1. The Cd concentration 20 mg/L used in this study is too high but could be compromised because authors selected it as one of the treatments to study Cd toxicity effects on maize varieties. However, the surprising thing is that this higher toxicity of Cd did not significantly affect the biomass of root or root shoot ratio in FY9 cultivar relative to control Table 2. Please explain the possibilities for these results?

Response: We think this is an excellent question. It is very important to select the appropriate concentration of Cd to treat samples in the experiment. In this study, we treated the seedlings with different concentrations of CdCl_2_, namely 5, 10, and 20 mg/L. Because the molecular weight of CdCl_2_ is 183.32, the molar concentration of 20 mg/L CdCl_2_ is about 100 μM/L. Because the treatment times and materials varied, the Cd concentrations in the treatment were different, and the Cd concentrations ranged from 1 μM/L to 500 μM/L. Other researchers have treated crops with Cd at high concentrations, for example, Verma et al. (2001) treated rice with 500 μM/L CdCl_2_ for 20 days and Ren et al. (2019) treated maize with 200 mg/L CdCl_2_•2.5H_2_O for 3 days. Therefore, we considered that a concentration of 20 mg/L CdCl_2_ was appropriate for treating the maize for 18 days.

In previous studies, we examined how 16 maize varieties that are widely grown in the northeast of China responded to Cd stress according to the changes of phenotype. We found that FY9 and SY33 represented low and high sensitivities varieties, respectively. The growth of FY9 was not significantly inhibited when Cd was added, but the biomass of the shoots and roots decreased by 28% and 15%, respectively. Liu et al. (2007) found that different accessions had different tolerances to Cd street. Also, different plant organs responded differently to Cd stress. For example, Khan et al. (2020) found that the dry weight of Brassica rapa ssp. chinensis L. (pak choi) leaves decreased by more than that of the roots. Therefore, the difference in the genotype meant that FY9 had low sensitivity to Cd stress, and, in this variety, more photosynthetic products were used to accumulate material rather than resist Cd stress when Cd was added, especially to roots.

2. Similarly most of the investigated parameters e.g. sucrose, fructose, glucose and similarly enzymatic activities were not significantly affected with higher Cd treatments relative to control in root tissues than shoot. Please could authors prove these results with some references that Cd toxicity has negligible effects on root relative to shoots in crop plants?

Response: We have not been able to find any references that directly prove that Cd toxicity has less effect on roots than shoots at the physiological level in the crops. We did however find that our results for changes in biomass were similar to those of other studies, and that the reductions in biomass were more pronounced in shoots of maize and Brassica rapa than in the roots under Cd stress (Abdelgawad et al., 2020, Khan et al., 2020). Different organs had different sensitivities to Cd stress in plants. Stress from heavy metals limits the nutrient uptake and affects the water transport from the roots to the shoots and upsets the water balance in plants (Schat et al., 1997). The shoots were more sensitive to water stress than the root so the sugar contents and enzymatic activities of sucrose metabolism in the shoots were more significantly affected than the roots, as they tried maintain the osmotic balance.

3. Introduction doesn’t end with specific objectives of the study?

Response: Line 88-92. We have added the objectives of the study in the introduction of the unmarked version.

4. Hoagland solution composition is not mentioned.

Response: Line 103-105. We have added information about the Hoagland solution composition in the materials and methods of the unmarked version.

5. Statistical analysis is not explained.

Response: We apologize for this omission. Line 167-171. We have added information about the statistical analysis to the materials and methods of the unmarked version.

6. Results are more general and based on well-established reports e.g. Pn decreased, sugar contents increased or Cd concentrations also increased with the increase of Cd treatments.

Response: Yes, the results of our study, such as the decreased Pn, increased sugar contents, and changes in enzyme activities as the Cd concentrations in the two varieties increased, are similar to those published elsewhere. However, we focused on the effects of Cd on sucrose metabolism and compared how the sucrose metabolism varied between maize varieties with low and high Cd sensitivities. Although the trends of sucrose metabolism were similar between two varieties, we found that there were more soluble sugars in Cd highly sensitive variety (SY33) than in FY3 and that the biomass was higher in low sensitivity variety (FY9) than in SY33. This shows that photosynthetic products are used to support maize growth in the low sensitivity varieties under Cd stress, but are used to defend Cd stress and maintain seedlings in highly sensitive varieties.

7. Discussion should explain the possible reasons for the determined parameters (increase or decrease) but this part is not properly elucidated, so this part should be rewritten with probable reasoning.

Response: Thank you for your suggestion. We have rewritten the discussion and explained the reasons for the changes in the parameters.

8. MS needs significant improvement regarding English language as there are lots of typographic or language mistakes that are difficult to highlight in present form.

Response: Thank you for your comment. We have modified and improved the English language and expression in the manuscript.

Reviewer #2:

1. " the soluble sugars that come from sucrose metabolism products have important roles as osmotic protectants and signal molecules that defend against Cd stress,"

What does this mean? --"sucrose metabolism may be a passive response " Why is passive? this is in need some explanation.

Response: We apologize for the ambiguity caused by the word “passive”. We have changed “passive” to “secondary” in lines 31 and 352, respectively. Plants have evolved many defense systems to cope with Cd stress, such as antioxidant systems and osmotic regulation systems. Antioxidant systems produce antioxidants and enzymes to remove the excess ROS induced by Cd stress. While the products of sucrose metabolism are mainly used for plant growth. When plants are subjected to severe osmotic stress, sucrose metabolites are used to maintain the osmotic balance in plants. Cd stress is more serious in highly sensitive maize varieties. That might mean that their defense systems are weak, such that they need more more sucrose metabolites to help plants survive under Cd stress. The situation is reversed in low sensitivity varieties of maize.

2. It would be more helpful to put in Cd-tolerant and Cd-sensitive cultivars rather than FY9 and SY33, respectively, in the Abstract, results and discussion.

Response: Thank you for your suggestion. We would like to explain this further. We used “FY9” and “SY33” to represent varieties with low and high sensitivities to Cd stress throughout the manuscript, respectively. Although the words “Cd-tolerant cultivars and Cd-sensitive cultivars” show more clearly the varieties response to Cd stress, they are very long and perhaps too long to use frequently throughout the paper. Hence, we use “Cd-tolerant and Cd-sensitive cultivars” instead of “FY9 and SY33” in the concluding sentence, and in lines 33, 199-200, 217.

3. In the methods and materials, there should be statistical analysis, and info on replicates.

Response: Thank you for your suggestion. We have added information about statistical analysis and the replicates to the materials and methods.

Editor’s suggestion

1. Avoid abbreviation in the title. Introduction section contains various unnecessary statements. Remain focused on the topic. Begin with the broadest scope and get progressively narrower, leading steadily to the statement of objectives. Be clear regarding objectives.

Response: Thank you for your suggestion. In the title, we have changed Cd to cadmium. We have added the aims of the study to the introduction of the unmarked version in line 88-92.

2. Material and Methods section: Several points need to be clarified. The information regarding statistical analysis and experimental design should be made clear.

Response: We have added information about the statistical analysis and Hoagland solution composition to the Material and Methods section in lines 167–171 and 103–105, respectively.

3. In results, better to add the numeric description of results (% variations).

Response: Thank you for your suggestion. We have added the numerical description of the results to the results section in lines 193–196, 207, and 214.

4. Discussion should be merely based on the observed findings. Not just a review of literature. Answer the question posed in introduction and correlate your finding with the existing knowledge.

Response: Thank you for your suggestion. We have rewritten the discussion and explained the reasons for the changes in the parameters. We have also answered the question in the introduction.

5. Language needs substantial improvement. There are several grammatical and typo mistakes throughout the manuscript.

Response: Thank you for your comment. We have modified and improved the English language of the manuscript.

6. Please remove any funding-related text from the manuscript and let us know how you would like to update your Funding Statement. Currently, your Funding Statement reads as follows:

"The funders had no role in study design, data collection and analysis, decision to publish, or preparation of the manuscript."

Response: We modified our Funding Statement as follows:

This research was financially supported by the scientific research projects of Liaoning Provincial Department of Education, China (No. LSNZD201608 and LSNYB201607). The funders had no role in study design, data collection and analysis, decision to publish, or preparation of the manuscript.

Abdelgawad H, Zinta G, Hamed BA, Selim S, Beemster GT, et al. Maize roots and shoots show distinct profiles of oxidative stress and antioxidant defense under heavy metal toxicity. Environmental Pollution. 2020; 258; 113705.

Khan KY, Ali B, Stoffella PJ, Cui X, Yang X, et al. Study amino acid contents, plant growth variables and cell ultrastructural changes induced by cadmium stress between two contrasting cadmium accumulating cultivars of Brassica rapa ssp. chinensis L. (pak choi). Ecotoxicology and Environmental Safety. 2020; 200; 110748.

Liu J, Qian M, Cai G, Yang J, Zhu Q. Uptake and translocation of cd in different rice cultivars and the relation with cd accumulation in rice grain. Journal of Hazardous Materials. 2007;143 (1-2): 443-447.

Ren, W., Liu, Y., Zhou, M., Shi, Z., Wang, T., Zhao, J., et al. Dynamic changes of root proteome reveal diverse responsive proteins in maize subjected to cadmium stress. Journal of Integrative Agriculture. 2019; 18(10); 2193-2204.

Schat, H., Sharma, S.S., Vooijs, R., 1997. Heavy metal-induced accumulation of free proline in a metal-tolerant and a nontolerant ecotype of Silene vulgaris, 101, 477e482.

Verma S, Dubey RS. Effect of cadmium on soluble sugars and enzymes of their metabolism in rice. Biologia Plantarum. 2001;44 (1): 117-123.
