# Supplementary material for: Changes in sucrose metabolism in maize varieties with different cadmium sensitivities under cadmium stress
Source: PLoS One. 2020 Dec 11;15(12):e0243835. doi: 10.1371/journal.pone.0243835 (PMC7732117; doi:10.1371/journal.pone.0243835)
Supplement: Attachment — Submitted filename: Response to review5.docx [file pone.0243835.s003.docx]

Dear Editor:

We thank the editor and reviewers for comments and suggestions about our article entitled “Changes in sucrose metabolism in maize varieties with different cadmium sensitivities under cadmium stress” (ID: PONE-D-20-20550). We have revised the manuscript according to your suggestions. The revised parts are marked in red in the paper. We have compiled the main corrections and the responses to the reviewers’ comments as below.

Reviewer #1: It is appreciated that authors have tried their best to improve the manuscript but still following are some concerns that must be addressed:

1.There are no line numbers in the present form of ms and so authors should use line number option as it is difficult to highlight the mistakes at specific points

Response: We apologize for the inconvenience caused by the omission. Line number have been added to the manuscript.

2.‘The seeds were sterilized in a 1% sodium hypochlorite solution (v/v) for 10 min and rinsed with sterile water three times and then soaked in sterile water at 4 °C for 24 h to adsorb water.’ Why seeds were soaked at such low temperature (4 °C) for 24h?

Response: In this experiment we thought that seeds imbibition in low temperature could increase the consistency of seed germination. But our other experiments showed that the uniform of seed germination in low temperature was similar to that in other temperatures, such as 20 or 28°C.

3.‘The germinated uniform seeds were transferred into plastic containers’ how old seedlings were transferred? should mention.

Response: We apologize for this omission. The seedlings germinated for 3 days were transferred into plastic containers. We have added the information in marked version line 103-104.

4. ¼ strength of Hoagland solution was applied for how many days and when full strength was started? Should be clear

Response: In our previous experiment, the 1/4 Hoagland solution could provide sufficient nutrients for seedling growth at the stage of this experiment. Therefore, it was applied for the whole cultivating and stress treatment stages.

5.‘The dry plant tissues were mixed with HNO3 and HClO4 (v/v, 83/17) for 24 h.’ how 83/17 ratio was maintained and it’s better to simply by division.

Response: We improved the Cd concentrations determination method according to the method of Guo et al (2019). The ratio of HNO3 and HClO4 (v/v, 83/17) is suitable for the extraction of Cd in our experiment.

6. Protein contents were measured by Bradford method but protein content data is not provided in the ms.
Response: Thank you for your comment. The content about protein content measure was removed from ms.

7. Please provide reference for RNA extraction and analysis methods used.

Response: RNA extraction was made using RNAprep pure Plant Kit (Qiagen). The reference for analysis method has been added in marked version line 163.

8. ‘These data suggest that Cd had an influence on photosynthesis in the maize seedlings and had more effect on Cd-sensitive variety than Cd-tolerant variety.’ Please specify positive or negative influence?

Response: Thank you for your suggestion. We have added the word “negative” in marked version line 201.

9. Somewhere used ‘sugar contents’ while somewhere ‘sugar concentration’ please be specific throughout ms

Response: Thank you for your suggestion. We have replaced “sugar concentration” with “sugar contents” in marked version line 207.

10.‘The expression of ZmSUT1 was higher in the shoots and roots of Cd-treated FY9, and expressions of ZmSUT1 and ZmSUT4 were higher in the shoots and roots of Cd-treated SY33, than in the control (Fig 6A, B, C, D).’ But ZmSUT1 expression was higher in shoots while decreased in roots in Cd-treated FY9 as compared to control. Please confirm this result and correct.

Response: We apologize for the inaccurate statement. The sentence has been modified to “The expression of ZmSUT1 was higher in the shoots under Cd-treated, while it was lower than control under 20 mg L^−1^ Cd-treated in the roots of FY9.The expressions of ZmSUT1 and ZmSUT4 were higher in the shoots and roots of Cd-treated SY33, than in the control (Fig 6A, B, C, D).” in the line marked version 253-255.

11. It was Cd concentration that was determined in shoots and root tissues but in fig legends and fig 1 it is mentioned Cd content. Please correct it throughout ms.

Response: Thank you for your suggestion. We have replaced “Cd content” with “Cd concentration” in marked version line 26, 383, 590, and Fig. 1, respectively.

12. Please explain the full names of SPS, AI, and SS in fig 4 legends

Response: Thank you for your suggestion. The full names of SPS, AI, and SS have been added to Fig. 4 legends in marked version line 606-607.

13.‘The total soluble sugar contents showed a clear increase when the Cd concentrations in the roots were low in the roots of the Cd-sensitive maize, SY33.’ Please revise it.

Response: Thank you for your suggestion. The sentence has been modified to “The total soluble sugar contents showed a clear increase when Cd concentrations were low in the roots of the Cd-sensitive maize, SY33.” In marked version line 321-322.

14. Please simplify this sentence ‘The AI and SS-H activities were higher in the shoots of SY33 than in those of FY9, and were higher in the shoots of both plants than in the control, as the soluble sugars in the shoots of both varieties increased as the Cd concentration increased.’

Response: Thank you for your suggestion. The sentence has been modified to “The AI and SS-H activities were higher in the shoots than in the control, and were higher in SY33 than that in FY9 as Cd concentration increased.” in marked version line 299-300.

15. I would suggest authors to thoroughly go through the whole ms to correct some typographic mistakes and take help from native speaker to improve English language. For example, there is a significant need to improve main headings and the word ‘THE’ is unnecessarily used throughout ms.

Response: Thank you for your suggestion. We have modified and improved the English language in the manuscript.

Reviewer #2: (No Response)

Reviewer #3: In the manuscript „Changes in sucrose metabolism in maize varieties with different cadmium sensitivities under cadmium stress”, the authors describe the effects of increasing cadmium concentrations on the seedlings of two different maize varieties. The topic is well presented and the experimental approach is correct. The results are properly described and are clear. However, the discussion is confusing and there is not a clear thread. Each of the results are rather discussed separately and not used to build up a round story. This is perfectly reflected in the last paragraph, where instead of summarizing the conclusion and go to the point, starts to divagate once more about the results. I strongly encourage the authors to re-write the discussion and be clear, highlight the main experiment, as shown by the title (impact of cadmium on sucrose metabolism) and use the rest to support it.

Response: Thank you for your comment and suggestion. We have modified the discussion to make it clearer. First, we investigate the phenotype of two varieties under Cd stress, and then used the changes of sucrose metabolism in different sensitive maize varieties, including the change of photosynthesis products, sucrose metabolism enzyme activities, sugar contents and the transportation and distribution of sugars, to explain the phenotype change. In the last paragraph, we illuminated the impact of cadmium on sucrose metabolism and dissected the mechanism by which sucrose metabolism response to Cd stress in two different sensitive maize varieties.
