# Supplementary material for: Changes in sucrose metabolism in maize varieties with different cadmium sensitivities under cadmium stress
Source: PLoS One. 2020 Dec 11;15(12):e0243835. doi: 10.1371/journal.pone.0243835 (PMC7732117; doi:10.1371/journal.pone.0243835)
Supplement: Attachment — Submitted filename: Response to review6.docx [file pone.0243835.s004.docx]

Dear Editor:

We thank the reviewers for comments and suggestions about our article entitled “Changes in sucrose metabolism in maize varieties with different cadmium sensitivities under cadmium stress” (ID: PONE-D-20-20550). We have revised the manuscript according to your suggestions. The revised parts are marked in red in the paper. We have compiled the main corrections and the responses to the reviewers’ comments as below.

Reviewer #1: It is appreciated that authors have incorporated suggestions/comments in the revised MS but still my concern is about statistical analysis:

The present study is comprised of two maize varieties and different Cd concentrations, so there are two independent factors but authors report at lines 167-168 that one way ANOVA was used for analyzing the collected data. So authors should have a carefully look that whether two way ANOVA was applicable? And how the interaction effects were compared and analyzed according to present statistical analysis? Although, authors have significantly improved English language but still I would suggest improving the structure/fluency of the sentences by some experts.

Response: Thank you for your suggestions. Indeed, two-way ANOVA is more applicable for this study. We have reanalyzed the data and interaction effects in two-way ANOVA. In addition, the unclear sentence has been revised. These results have been added in modified version.

Reviewer #3: The authors have re-written the discussion and now it is improved. Just some minor things:

Sentence in l. 280 needs rewording, the sentence is not very clear.

Response: I apologize for this omission. We have reworded the sentence in modified version.

l. 259, a space after stop is missing

Response: Space has been added in modified version.

l. 262 Brassica rapa should be written in italics

Response: Brassica rapa has been written in italics in modified version.
